# Infant and young child feeding practices and anthropometric outcomes among children under 2 years in the Northern Emirates of the UAE: an analytical cross-sectional study

**DOI:** 10.3389/fnut.2026.1890340

**Published:** 2026-07-24

**Authors:** Mohamed Anas Patni, Moza Saif Hassan Ramadhan Almarzooqi, Fatima Alzahraa Adnan Alhussein, Maryam Mohammed Saeed Mansoor Almansoori, Naeema Saeed Mohamed, Mariam Mohamed Abdalla Mohamed Alrayssi, Rasha Aziz Attia Salama, Subhranshu Sekhar Kar, Rajani Dube, Biji Thomas George, Aamenah Barakat, Mushirabanu Sharifmiyan Akikwala

**Affiliations:** 1 Department of Community Medicine, RAK College of Medical Sciences, Ras Al Khaimah Medical and Health Science University, Ras Al Khaimah, United Arab Emirates; 2 Research Unit, Center for Health Work Force Development, Ras Al Khaimah Medical and Health Science University, Ras Al Khaimah, United Arab Emirates; 3 Department of Community Medicine, Kasr El Aini Faculty of Medicine, Cairo University, Giza, Egypt; 4 Department of Pediatrics, RAK College of Medical Sciences, Ras Al Khaimah Medical and Health Science University, Ras Al Khaimah, United Arab Emirates; 5 Department of Obstetrics and Gynecology, RAK College of Medical Sciences, Ras Al Khaimah Medical and Health Science University, Ras Al Khaimah, United Arab Emirates; 6 Department of General Surgery, RAK College of Medical Sciences, Ras Al Khaimah Medical and Health Science University, Ras Al Khaimah, United Arab Emirates; 7 Department of Pediatrics, Saqr Hospital, Emirates Health Services, Ras Al Khaimah, United Arab Emirates; 8 Department of Pediatrics, Shifa Al Jazeera Medical Center, Ras Al Khaimah, Ras Al Khaimah, United Arab Emirates

**Keywords:** exclusive breastfeeding, infant and young child feeding (IYCF), length-for-age Z-score (LAZ), minimum acceptable diet, minimum dietary diversity (MDD), minimum meal frequency, pre-lacteal feeding (PLF), weight-for-age z-score (WAZ)

## Abstract

**Background:**

Optimal infant and young child feeding (IYCF) practices are important determinants of early childhood growth and development. Although breastfeeding and complementary feeding recommendations are well established, gaps often remain between maternal knowledge and actual feeding practices. Local evidence from the Northern Emirates of the United Arab Emirates (UAE) is limited, particularly regarding differences between local and expatriate mothers and the relationship between IYCF practices and child anthropometric outcomes. This study assessed maternal knowledge and practices related to IYCF and examined their association with weight-for-age (WAZ) and length-for-age z-scores (LAZ) among children under 2 years in Ras Al Khaimah and Fujairah, UAE.

**Methods:**

An analytical cross-sectional study was conducted among 381 mother–child pairs with children aged up to 24 months. Participants were recruited from community settings and pediatric outpatient areas. Data were collected using a structured English and Arabic questionnaire, and child anthropometric measurements were recorded. Descriptive statistics, chi-square tests, independent-samples *t*-tests, and regression analysis were used.

**Results:**

The mean maternal age was 29.14 ± 6.53 years, and the mean child age was 15.04 ± 7.22 months. Knowledge was generally high for early initiation of breastfeeding, colostrum feeding, and exclusive breastfeeding, but significant differences between local and expatriate mothers were observed for selected knowledge indicators. Correct early initiation of breastfeeding was reported by 284 mothers (74.5%), while exclusive breastfeeding for 6 months was reported by 189 of 345 eligible mothers (54.8%). Among eligible children aged 6–23 months, minimum dietary diversity, minimum meal frequency, and minimum acceptable diet were achieved by 163 (47.2%), 185 (53.6%), and 134 (38.8%) children, respectively. In adjusted linear regression, early initiation of breastfeeding, exclusive breastfeeding, and higher maternal knowledge score were positively associated with WAZ, while number of children was negatively associated with WAZ. Exclusive breastfeeding was positively associated with LAZ, whereas male sex was negatively associated with LAZ.

**Conclusion:**

Maternal knowledge of recommended IYCF practices was generally favorable, but important gaps remained in actual feeding practices, particularly exclusive breastfeeding, avoidance of pre-lacteal feeding, continued breastfeeding, and minimum acceptable diet. As this was a cross-sectional study, the observed relationships between IYCF practices and anthropometric outcomes should be interpreted as associations rather than causal effects. Strengthening culturally appropriate breastfeeding and complementary feeding counseling may help improve recommended IYCF practices in the Northern Emirates of the UAE.

## 1 Introduction

Infant and young child feeding is a major determinant of child survival, growth, development, and long-term health (1). The first 2 years of life are a critical period during which nutritional exposures influence physical growth, immune function, neurodevelopment, and later risk of non-communicable diseases (1, 2). The first 1,000 days, from conception to 2 years of age, are also recognized as a critical window for growth, neurodevelopment, immune development, and later health outcomes (2, 3). Appropriate feeding during this period includes initiation of breastfeeding within the 1 h of birth, exclusive breastfeeding for the first 6 months, timely introduction of safe and adequate complementary foods from 6 months and continued breastfeeding up to 2 years or beyond (1, 4).

Early initiation of breastfeeding provides the newborn with colostrum and supports early immune protection, while delayed initiation may increase exposure to infectious pathogens and interfere with breastfeeding establishment (1, 5). Breastfeeding has well-documented short- and long-term benefits, including protection against infectious morbidity in childhood and potential protection against later overweight, obesity, and metabolic risk (5, 6). Exclusive breastfeeding for the first 6 months provides adequate nutrition and reduces the risk of diarrhea and respiratory infections, while pre-lacteal feeding and early formula feeding may increase infection risk and disrupt optimal breastfeeding practices (1, 5, 7). From 6 months onwards, breast milk alone becomes insufficient to meet nutritional needs, and safe, age-appropriate complementary feeding is required to prevent undernutrition, micronutrient deficiencies, and growth faltering (4, 8). Inadequate dietary diversity, insufficient meal frequency, and failure to achieve minimum acceptable diet during 6–23 months may compromise growth and increase vulnerability to morbidity (4, 8).

IYCF practices are influenced by maternal knowledge, cultural beliefs, family support, socioeconomic conditions, maternal employment, healthcare access, and the quality of counseling received during antenatal, delivery, and postnatal care (1, 9). Previous evidence from South Gujarat has shown that poor IYCF practices are significantly associated with higher morbidity episodes, particularly diarrhea and acute respiratory tract infections, among children up to 24 months (7). Another cohort study among children with severe acute malnutrition reported that children's weight improvement was linked to better IYCF practices, highlighting the importance of caregiver education, exclusive breastfeeding, continued breastfeeding, and appropriate complementary feeding (9). These findings suggest that IYCF is not only nutritional behavior but also an important public health exposure related to infection, growth, and recovery from malnutrition (7, 8).

The Middle East and North Africa region is experiencing a nutrition transition, with undernutrition, micronutrient deficiencies, overweight, obesity, and diet-related non-communicable diseases coexisting within the same populations (10). UNICEF has also highlighted that children and women in the MENA region face multiple and overlapping forms of malnutrition, indicating the need for context-specific nutrition strategies (10). In the UAE, evidence from the Feeding Infants and Toddlers Study 2020 showed a double burden of malnutrition among infants and toddlers aged 0–23.9 months, supporting the need for improved early feeding practices and nutritional surveillance (11). A study from selected Northern Emirates of the UAE reported that research on infant and toddler feeding practices in the UAE remains limited and found that only 46.7% of infants were exclusively breastfed during the first 6 months (12). Previous UAE studies have provided important evidence on infant and toddler feeding practices and nutritional status. However, limited UAE evidence has specifically compared IYCF knowledge and practices between local and expatriate mothers and examined the association of individual IYCF practices with anthropometric z-scores such as WAZ and LAZ.

Therefore, the present study aimed to assess maternal knowledge and practices regarding breastfeeding and complementary feeding among mothers of children up to 24 months of age in Ras Al Khaimah and Fujairah. The study also compared selected IYCF indicators between local and expatriate mothers and examined their associations with weight-for-age and length-for-age z-scores.

## 2 Materials and methods

### 2.1 Study design

This analytical cross-sectional study was conducted among mother–child pairs in Ras Al Khaimah and Fujairah, United Arab Emirates (UAE). The study examined maternal infant and young child feeding (IYCF) knowledge and practices as exposure variables and child anthropometric indices as outcome variables. Because exposure and outcome data were collected at the same point in time, the findings were interpreted as associations rather than causal effects.

### 2.2 Study population and sample size

The study population comprised local Emirati and expatriate mothers with children aged up to 24 months residing in or attending selected study sites in the Northern Emirates of the UAE. Participants were recruited from local and expatriate communities in Ras Al Khaimah and Fujairah, as well as from pediatric outpatient waiting areas of Emirates Health Services hospitals in Ras Al Khaimah.

Mothers with children aged up to 24 months who provided informed consent and were willing to participate were included. Mothers of children aged 2 years and above were excluded from reducing recall bias related to early feeding practices. Mothers of children with chronic diseases, inborn errors of metabolism, or physical defects were excluded because these conditions could influence feeding patterns and anthropometric outcomes. Mothers attending emergency departments or whose children were admitted as inpatients were also excluded because acute illness or hospitalization could affect feeding practices and anthropometric interpretation. Delivery term was recorded as full term or preterm based on maternal report. Preterm birth was defined as birth before 37 completed weeks of gestation. Delivery term was used for descriptive purposes only. However, exact gestational age, corrected age for preterm children, and birth weight were not available from clinical records. Therefore, delivery term was used for descriptive purposes only and was not included as an adjustment variable in the multivariable anthropometric models.

For sample size estimation, the ideal target population was mothers with children under two years in Ras Al Khaimah and Fujairah. However, because precise local estimates for this subgroup were not available, the population of children under 15 years was used as a proxy to estimate the finite population size. According to World Bank data, children aged 0–14 years constituted approximately 15% of the UAE population. (13) The estimated population of Ras Al Khaimah in 2021 was approximately 0.4 million (14), while the estimated population of Fujairah was 256,256 in 2019 (15). Considering 15% of the combined population of approximately 0.65 million, the estimated child population in Ras Al Khaimah and Fujairah was approximately 97,500. Using this as the population estimate and using 46.7% as the anticipated proportion of mothers practicing exclusive breastfeeding based on a previous UAE study by Ali et al. (12), the sample size was calculated using the single population proportion formula with finite population correction:


$$n\;=\;\left[N\;\times \;Z^{2}\;\times \;p\left(1\;-\;p\right)\right]/\left[d^{2}\;\times \;\left(N\;-\;1\right)+Z^{2}\times p\left(1-p\right)\right]$$ (1)

where *n* is the required sample size, *N* is the estimated finite population size, *Z* is the standard normal value for 95% confidence level, *p* is the anticipated proportion, and d is the absolute precision. The assumptions used were: *N* = 97,500, Z = 1.96, *p* = 0.467, based on a previous UAE study reporting exclusive breastfeeding (12), and *d* = 0.05. Using these assumptions, the required sample size was approximately 381. Therefore, 381 mother–child pairs were included. Although this population estimate does not exactly represent mothers with children under two years, it provided a conservative and feasible basis for sample size calculation in the absence of more specific local data. Non-probability consecutive sampling was used because of feasibility constraints and the need to recruit eligible mother–child pairs from both community and outpatient settings. Efforts were made to include participants from both Ras Al Khaimah and Fujairah and to recruit both local and expatriate mothers.

### 2.3 Data collection tool and procedure

The questionnaire was developed after reviewing WHO/UNICEF IYCF indicators and relevant published literature on breastfeeding, complementary feeding, and child nutritional outcomes. The draft questionnaire was reviewed by experts in community medicine, pediatrics, and maternal and child health to assess content relevance, clarity, and cultural appropriateness. The tool was prepared in English and Arabic, and the Arabic version was reviewed by bilingual experts to ensure conceptual equivalence with the English version. The questionnaire was pilot tested among mothers similar to the target population, and minor wording changes were made for clarity before final data collection. Pilot responses were not included in the final analysis.

Internal consistency was explored using Cronbach's alpha. The alpha value was 0.46 for the knowledge items after excluding one constant item and 0.55 for the practice indicators. These values were interpreted cautiously because the items represented distinct IYCF indicators rather than a single latent construct. Therefore, content validity, expert review, bilingual review, and pilot testing were considered more relevant for assessing the suitability of the questionnaire. The findings were interpreted primarily at the level of individual IYCF indicators rather than as a single unified scale.

The questionnaire included three main sections. The first section collected sociodemographic information, including maternal age, child age, nationality status, emirate, maternal education, monthly family income, place of delivery, number of siblings, birth order, maternal working status, delivery term, and type of delivery. The second section assessed maternal knowledge and practices related to IYCF. Knowledge questions covered recommended timing of breastfeeding initiation, pre-lacteal feeding, colostrum feeding, exclusive breastfeeding, timing of complementary feeding, continued breastfeeding, and bottle feeding. Practice questions assessed actual timing of breastfeeding initiation, reasons for delayed initiation, pre-lacteal feeding, colostrum feeding, exclusive breastfeeding for the first 6 months, formula feeding, current breastfeeding, timing of complementary feeding, dietary diversity, meal frequency, consumption of selected food groups, sweet beverages and unhealthy foods, and bottle feeding. Knowledge items were scored as 1 for a correct response and 0 for an incorrect or ‘don't know' response. The total knowledge score was calculated by summing the item scores, with higher scores indicating better IYCF knowledge. Practice indicators were similarly coded as 1 for recommended practice and 0 for non-recommended practice. The total practice score was calculated by summing the coded practice items, with higher scores indicating better adherence to recommended IYCF practices. The third section included anthropometric measurements. The draft tool was reviewed by experts in community medicine and pediatrics for content relevance and clarity. The questionnaire was prepared in English and Arabic, and the Arabic version was reviewed for conceptual equivalence.

Eligible mothers were recruited consecutively from two types of study settings: community locations in Ras Al Khaimah and Fujairah and pediatric outpatient waiting areas of Emirates Health Services hospitals in Ras Al Khaimah. However, recruitment source was not recorded as a separate variable; therefore, the exact number and percentage of participants recruited from community versus outpatient settings could not be reported. Community recruitment was conducted through accessible public/community locations where mothers of young children were available, including maternal and child health–related community contacts and local family/community networks. In pediatric outpatient settings, mothers accompanying children under 24 months were approached while waiting for consultation. All mothers were screened for eligibility, the study purpose was explained, and written informed consent was obtained before participation. Mothers who declined participation or did not meet the eligibility criteria were not included. The number of eligible mothers approached and the number who declined participation were not recorded systematically. Therefore, the overall response rate could not be calculated. The purpose of the study was explained, and written informed consent was obtained before participation. Anthropometric measurements were collected by trained data collectors using standardized procedures. Weight was measured in kilograms using a calibrated weighing scale, with the child wearing light clothing and without shoes. The scale was checked regularly during data collection. For children under 24 months, recumbent length was measured using an infantometer or length board, with the child positioned supine and the head and feet properly aligned. Where a measurement appeared inconsistent or the child was moving excessively, the measurement was repeated and the more reliable value was recorded. Nutritional outcomes included weight-for-age z-score and length-for-age z-score, which were used to compare mean anthropometric status between children with correct and incorrect feeding practices. These indices were calculated using WHO Anthro software, based on the WHO Child Growth Standards. The child's age, sex, weight, and recumbent length were entered into the software to generate weight-for-age z-scores (WAZ) and length-for-age z-scores (LAZ). Implausible WAZ and LAZ values generated by WHO Anthro were checked against the original anthropometric records and data-entry sheets. Data was collected from the eligible participants between October 2024 to March 2025.

### 2.4 Operational definitions

Early initiation of breastfeeding was defined as putting the child to the breast within 1 h of birth. Exclusive breastfeeding was defined as feeding the infant only breast milk for the first 6 months of life, without other foods or fluids, except oral rehydration solution, drops, syrups, vitamins, minerals, or medicines when required. Pre-lacteal feeding referred to giving any food or fluid before initiation of breastfeeding. Colostrum feeding referred to the child receiving the first breast milk after birth. Timely introduction of complementary feeding was defined as introduction of solid, semi-solid, or soft foods at 6–8 months of age. Continued breastfeeding was assessed among children aged 12–23 months (16).

Minimum dietary diversity (MDD) was defined as the proportion of children aged 6–23 months who consumed foods and beverages from at least five of eight food groups during the previous day, including breast milk as one of the food groups. Minimum meal frequency (MMF) was defined according to age and breastfeeding status. Minimum acceptable diet (MAD) was defined as meeting both minimum dietary diversity and minimum meal frequency criteria. Bottle feeding referred to feeding the child using a bottle with nipple/teat. For analysis, IYCF knowledge and practice indicators were classified as correct or incorrect according to recommended feeding practices. Weight-for-age z-score and length-for-age z-score were treated as anthropometric outcome indicators (16).

### 2.5 Ethical issues

The study was reviewed and approved by the Research Ethics Committee of Ministry of Health and Prevention, Ras Al Khaimah with the approval number No. MOHAP/REC/2024/106-2024-F-M. The study was conducted in accordance with institutional ethical requirements. Written informed consent was obtained from all participating mothers before data collection.

### 2.6 Data analysis

Data was entered into Microsoft Excel and analyzed using IBM SPSS Statistics version 30. Data were checked for completeness, coding errors, and out-of-range values before analysis. No missing data were identified in the final dataset; therefore, all 381 participants were included in the main descriptive and comparative analyses, except for age-specific indicators such as exclusive breastfeeding and complementary feeding indicators, where the denominator was restricted to eligible children. Descriptive statistics were used to summarize participant characteristics. Frequencies and percentages were used for categorical variables, while means and standard deviations were used for continuous variables. Normality of continuous variables was assessed using visual inspection of histograms and Q–Q plots. Anthropometric z-scores, including WAZ and LAZ, showed approximately normal distributions and were treated as continuous outcome variables. Knowledge and practice scores were bounded summary scores and were interpreted descriptively; where mean comparisons were performed, findings were interpreted cautiously in view of their score-based nature and the sample size. Chi-square tests were used to assess differences in knowledge and practice indicators between local and expatriate mothers. Independent-samples *t*-tests were used to compare mean knowledge and practice scores and to compare mean anthropometric z-scores between children with correct and incorrect feeding practices.

Multivariable linear regression was performed separately for WAZ and LAZ as continuous outcome variables. Candidate predictors were selected based on biological plausibility, study objectives, and previous literature rather than only univariate statistical significance. Variables considered for model building included child age, child sex, nationality status, maternal education, maternal employment, family income sufficiency, number of children, total knowledge score, early initiation of breastfeeding, colostrum feeding, exclusive breastfeeding, pre-lacteal feeding, complementary feeding practice, and bottle feeding. Final models retained variables that were clinically or epidemiologically relevant and contributed meaningfully to model fit. Multicollinearity was assessed using variance inflation factor values. Regression coefficients are reported as unstandardized B coefficients with 95% confidence intervals. Statistical significance was set at *p* < 0.05.

As an exploratory secondary analysis, binary logistic regression was performed for complementary feeding outcomes among eligible children aged six months and above. The outcomes were minimum dietary diversity (MDD), minimum meal frequency (MMF), and minimum acceptable diet (MAD), coded as met versus not met. Candidate predictors included nationality status, child age, maternal education, maternal working status, family income sufficiency, number of family members, number of siblings, maternal knowledge score, cesarean delivery, and prematurity. Adjusted odds ratios with 95% confidence intervals were reported.

## 3 Results

A total of 381 mother–child pairs were included. The mean maternal age was 29.14 ± 6.53 years and the mean child age was 15.04 ± 7.22 months. Most participants were local mothers, from Ras Al Khaimah, housewives, and had delivered full-term infants vaginally. Detailed sociodemographic characteristics are presented in Table 1.

**Table 1:** Sociodemographic characteristics of study participants.

Characteristic	Category	Frequency	Percentage
Nationality status	Local	216	56.7
	Expatriate	165	43.3
Emirate	Ras Al Khaimah	243	63.8
	Fujairah	138	36.2
Maternal education	Bachelor's degree	177	46.5
	High school	121	31.8
	Postgraduate	18	4.7
	Diploma	17	4.5
	Primary	15	3.9
	Illiterate	15	3.9
	Graduate	14	3.7
	PhD	4	1.0
Family income	Enough	185	48.6
	Partially enough	154	40.4
	Not enough	42	11.0
Place of delivery	EHS hospital	286	75.1
	Private hospital	92	24.1
	Home	3	0.8
Working status	Housewife	221	58.0
	Working	160	42.0
Delivery term	Full term	347	91.1
	Preterm	34	8.9
Type of delivery	Vaginal	285	74.8
	Cesarean section	96	25.2

Knowledge regarding recommended IYCF practices was generally high among both local and expatriate mothers as shown in Table 2. Significant differences between local and expatriate mothers were observed for knowledge of pre-lacteal feeding, colostrum feeding, and exclusive breastfeeding for the first 6 months. The mean knowledge score was 5.91 ± 1.16 among local mothers and 6.11 ± 1.10 among expatriate mothers; this difference was not statistically significant.

**Table 2:** Knowledge of infant and young child feeding practices among study participants.

Knowledge indicator	Local correct *n* (%)	Local incorrect *n* (%)	Expatriate correct *n* (%)	Expatriate incorrect *n* (%)	*p*-value
Timing of initiation of breastfeeding after birth	193 (89.4)	23 (10.6)	145 (87.9)	20 (12.1)	0.77
Pre-lacteal feeding	206 (95.4)	10 (4.6)	145 (87.9)	20 (12.1)	0.0079^*^
Colostrum feeding	201 (93.1)	15 (6.9)	165 (100.0)	0 (0.0)	< 0.001^*^
Exclusive breastfeeding for first 6 months	171 (79.2)	45 (20.8)	150 (90.9)	15 (9.1)	0.0016^*^
Timing of initiation of complementary foods	167 (77.3)	49 (22.7)	129 (78.2)	36 (21.8)	0.93
Continued breastfeeding in children aged 12–23 months	176 (81.5)	40 (18.5)	125 (75.8)	40 (24.2)	0.21
Bottle feeding recommended	158 (73.1)	58 (26.9)	115 (69.7)	50 (30.3)	0.55

^*^Statistically significant at *p* < 0.05.

Overall feeding practices were similar between local and expatriate mothers for most indicators as seen in Table 3. Correct timing of complementary feeding was significantly higher among expatriate mothers than local mothers, 76.1% vs. 63.2%, *p* = 0.009. The mean practice score was 3.01 ± 1.26 among local mothers and 3.05 ± 1.28 among expatriate mothers, with no significant difference, *p* = 0.76.

**Table 3:** Feeding practices among study participants.

Feeding practice indicator	Local correct*n* (%)	Local incorrect *n* (%)	Expatriate correct *n* (%)	Expatriate incorrect *n* (%)	*p-*value
Timing of initiation of breastfeeding after birth	159 (73.6)	57 (26.4)	125 (75.8)	40 (24.2)	0.72
Pre-lacteal feeding	114 (52.8)	102 (47.2)	85 (51.5)	80 (48.5)	0.89
Colostrum feeding	184 (85.2)	32 (14.8)	150 (90.9)	15 (9.1)	0.13
Exclusive breastfeeding for first 6 months	97 (51.1)	93 (48.9)	92 (59.4)	63 (40.6)	0.15
Timing of initiation of complementary foods	120 (63.2)	70 (36.8)	118 (76.1)	37 (23.9)	0.009^*^
Continued breastfeeding in children aged 12–23 months	35 (25.7)	101 (74.3)	38 (36.2)	67 (63.8)	0.079
Bottle feeding	85 (39.4)	131 (60.6)	70 (42.4)	95 (57.6)	0.53

**Figure 1:**
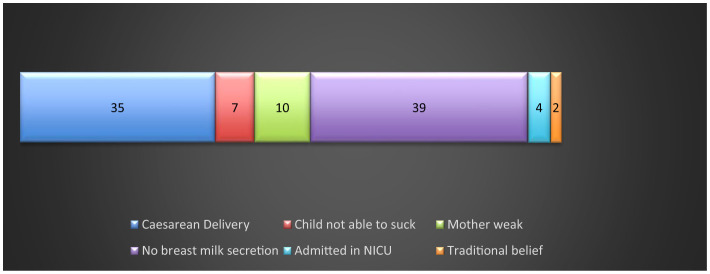
Reasons quoted by study participants for not starting Breast Feeding within 1 h (*n* = 97).

Among mothers who did not initiate breastfeeding within one hour of birth, the most commonly reported reasons were perceived absence of breast milk secretion, reported by 40.2%, and cesarean delivery, reported by 36.1%. Other reasons included maternal weakness, the child being unable to suck, neonatal intensive care admission, and traditional beliefs as shown in Figure 1.

Among the 156 children who were not exclusively breastfed during the first 6 months, 124 (79.5%) received formula feeding and 32 (20.5%) did not receive formula feeding.

Among eligible children aged 6–23 months, minimum dietary diversity was achieved by 163 children, 47.2%, minimum meal frequency by 185 children, 53.6%, and minimum acceptable diet by 134 children, 38.8%. When stratified by nationality status, MDD was achieved by 45.8% of local children and 49.0% of expatriate children. MMF was achieved by 54.7% of local children and 52.3% of expatriate children, while MAD was achieved by 37.9% and 40.0%, respectively. No statistically significant differences were observed between local and expatriate children for MDD, MMF, or MAD. The results are represented in Table 4.

**Table 4:** Minimum dietary diversity, minimum meal frequency, and minimum acceptable diet among eligible children aged 6–23 months by nationality status, *n* = 345.

Indicator	Local yes *n* (%)	Local no *n* (%)	Expatriate yes *n* (%)	Expatriate no *n* (%)	*p*-value
Minimum dietary diversity, MDD	87 (45.8)	103 (54.2)	76 (49.0)	79 (51.0)	0.56
Minimum meal frequency, MMF	104 (54.7)	86 (45.3)	81 (52.3)	74 (47.7)	0.63
Minimum acceptable diet, MAD	72 (37.9)	118 (62.1)	62 (40.0)	93 (60.0)	0.74

As an exploratory secondary analysis, binary logistic regression was performed for complementary feeding outcomes among eligible children aged 6 months and above. The models included nationality status, child age, maternal education, maternal working status, family income sufficiency, number of family members, number of siblings, maternal knowledge score, cesarean delivery, and prematurity as candidate predictors. Current breastfeeding was not included as an independent predictor because it forms part of the updated minimum dietary diversity definition. In the final models, higher child age and number of siblings were significantly associated with minimum dietary diversity. For minimum meal frequency, significant predictors were nationality status, child age, maternal working status, and family income sufficiency. For minimum acceptable diet, significant predictors were nationality status, maternal working status, and family income sufficiency. These findings are presented in Table 5.

**Table 5:** Exploratory logistic regression analysis of significant predictors of MDD, MMF, and MAD among eligible children aged six months and above.

Outcome	Significant predictor	Adjusted OR	95% CI	*p*-value
MDD	Child age, per month increase	0.94	0.90–0.98	0.004
	Number of siblings	1.27	1.00–1.61	0.047
MMF	Local nationality status	1.96	1.13–3.42	0.018
	Child age, per month increase	1.16	1.11–1.21	< 0.001
	Maternal working status	1.92	1.06–3.48	0.031
	Family income reported as enough	0.51	0.28–0.93	0.028
MAD	Local nationality status	2.27	1.12–4.58	0.022
		Maternal working status	2.59	1.26–5.33	0.010
		Family income reported as enough	0.46	0.22–0.96	0.039

Children with correct early initiation of breastfeeding had significantly higher mean weight-for-age z-scores than those with incorrect practice. Higher mean weight-for-age z-scores were also observed among children who received colostrum and those who were exclusively breastfed for the first 6 months. Pre-lacteal feeding, appropriate age of complementary feeding, continued breastfeeding among children aged 12–23 months, and bottle feeding were not significantly associated with weight-for-age z-score as shown in Table 6.

**Table 6:** Differences in mean WAZ scores among children by correct vs. incorrect feeding practices.

IYCF practice	Incorrect practice, mean ±SD	Correct practice, mean ±SD	*p*-value
Initiation of breastfeeding	1.21 ± 1.57	1.72 ± 1.56	0.0222^*^
Pre-lacteal feeding	1.48 ± 1.58	1.58 ± 1.57	0.654
Colostrum given	1.22 ± 1.51	1.69 ± 1.58	0.0327^*^
Exclusive breastfeeding given	1.19 ± 1.57	1.76 ± 1.57	0.011^*^
Appropriate age of complementary feeding	1.43 ± 1.47	1.56 ± 1.61	0.5517
Children aged 12–23 months currently breastfed	1.42 ± 1.60	1.67 ± 1.53	0.2601
Bottle feeding given	1.54 ± 1.55	1.51 ± 1.60	0.893

^*^Statistically significant at *p* < 0.05.

For length-for-age z-score, significant differences were observed for colostrum feeding and exclusive breastfeeding. Children who received colostrum and those who were exclusively breastfed for 6 months had higher mean LAZ than their counterparts. Other feeding practices were not significantly associated with LAZ scores. The findings are evident in Table 7.

**Table 7:** Differences in mean LAZ scores among children by correct vs. incorrect feeding practices.

IYCF practice	Incorrect practice, mean ±SD	Correct practice, mean ±SD	*p*-value
Initiation of breastfeeding	0.60 ± 1.00	0.79 ± 1.16	0.2163
Pre-lacteal feeding	0.68 ± 1.10	0.63 ± 1.15	0.7537
Colostrum given	0.58 ± 1.06	0.95 ± 1.13	0.0179^*^
Exclusive breastfeeding given	0.56 ± 1.10	0.94 ± 1.14	0.0174^*^
Appropriate age of complementary feeding	0.62 ± 1.11	0.72 ± 1.13	0.5286
Children aged 12–23 months currently breastfed	0.51 ± 1.07	0.76 ± 1.18	0.1181
Bottle feeding given	0.58 ± 1.12	0.70 ± 1.13	0.4516

^*^Statistically significant at *p* < 0.05.

The multivariable regression models explored the association of selected maternal, child, household, and IYCF-related variables with anthropometric outcomes. Variables considered for model building included nationality status, number of family members, maternal education, maternal working status, family income sufficiency, birth order, age and gender of the child, maternal knowledge score, breastfeeding practice, colostrum feeding, pre-lacteal feeding, complementary feeding practice, and bottle feeding. For Weight for age Z score, early initiation of breastfeeding, exclusive breastfeeding, and total knowledge score were positively associated, while number of children was negatively associated. For length for age Z score, exclusive breastfeeding was positively associated, while male sex was negatively associated. Details are presented in Table 8.

**Table 8:** Multivariable linear regression models for predictors of WAZ and LAZ.

Outcome	Predictor variable	B coefficient^*^	*t* statistic	*p*-value	95% CI
WAZ	Constant	0.77	2.08	0.04	0.15 to 0.97
	Initiation of breastfeeding within 1 h	0.71	2.99	< 0.001	0.24 to 1.16
	Exclusive breastfeeding	0.58	2.34	0.003	0.26 to 1.05
	Total knowledge score	0.22	2.13	0.03	0.02 to 0.39
	Number of children	−0.39	−2.08	0.04	−0.72 to −0.19
LAZ	Constant	0.83	1.58	0.114	−0.20 to 1.87
		Child's gender (boy vs. girl)	−0.213	−1.92	0.048	−0.440 to −0.0032
		Exclusive breastfeeding	0.49	2.21	0.021	0.09 to 1.05

^*^Regression coefficients are unstandardized B coefficients. Final model sample size for both models, *n* = 345. The adjusted R^2^ values were 0.11 for the WAZ model and 0.06 for the LAZ model. All VIF values were below 2.0, indicating no significant multicollinearity

## 4 Discussion

This study assessed maternal knowledge and practices related to infant and young child feeding among local and expatriate mothers in the Northern Emirates of the UAE and examined their association with anthropometric outcomes in children under 2 years. The findings show that maternal knowledge of recommended IYCF practices was generally high, but actual feeding practices were less consistent. Colostrum feeding and early breastfeeding were commonly reported, whereas exclusive breastfeeding for 6 months, avoidance of pre-lacteal feeding, continued breastfeeding during the 2 year of life, and minimum acceptable diet remained suboptimal. This pattern suggests that awareness alone may not be sufficient to ensure optimal feeding behavior. Although expatriate mothers had slightly higher mean knowledge scores than local mothers, mean practice scores were similar between the two groups. This finding suggests that knowledge alone may not be sufficient to ensure optimal feeding behavior. Practical barriers such as perceived insufficient milk, cesarean delivery, family influence, return to work, formula availability, and cultural beliefs may limit the translation of knowledge into practice in both groups. In the UAE's multicultural setting, expatriate mothers may differ in sources of health information and prior feeding norms, but both local and expatriate mothers may experience similar postnatal and household-level barriers (12).

The breastfeeding findings are broadly consistent with earlier UAE studies showing that recommended breastfeeding practices remain difficult to sustain. Radwan, in a study of Emirati mothers in the UAE, reported that mixed feeding was common and that many mothers introduced formula, liquids, or solids early in infancy (17). The same study highlighted the multiethnic and multicultural context of the UAE as an important factor contributing to variation in breastfeeding practices (17). More recent UAE evidence from the Feeding Infants and Toddlers Study also showed continuing concerns regarding infant and toddler feeding adequacy and nutritional status in children aged 0–23.9 months (5). Compared with these earlier UAE findings, the present study shows relatively favorable knowledge and early breastfeeding indicators, but continued challenges in maintaining exclusive breastfeeding and avoiding pre-lacteal or formula feeding.

Complementary feeding indicators in the present study also require attention. Minimum dietary diversity was achieved by 47.2% of eligible children, minimum meal frequency by 53.6%, and minimum acceptable diet by 38.8%. These findings should be compared cautiously with previous studies because the present study used the WHO/UNICEF 2021 definition of MDD, requiring consumption from at least five of eight food groups, including breast milk, whereas some earlier studies used the previous definition of at least four of seven food groups. For example, Taha et al. (18) reported that 71.4% of children consumed at least four food groups, 47.3% met minimum meal frequency, and 36.2% achieved minimum acceptable diet. However, the fact that more than half of children in the current study still did not meet minimum acceptable diet criteria indicates that complementary feeding quality remains an important area for intervention, even in settings where food availability may not be the main constraint.

Regional comparisons further show that suboptimal complementary feeding is not unique to the UAE. In a national study from Lebanon, Naja et al. reported that 92.8% of children aged 6–23 months met minimum meal frequency, but only 37.5% met minimum dietary diversity and 34.4% met minimum acceptable diet; the study also reported frequent consumption of unhealthy foods and sweet beverages (19). A Kuwait study using WHO IYCF indicators also examined feeding indicators in relation to anthropometric measurements, emphasizing the importance of monitoring IYCF practices in Gulf settings (20). These regional findings support the interpretation that complementary feeding programs should focus not only on whether food is given, but also on diversity, quality, frequency, and avoidance of unhealthy foods.

The association between feeding practices and anthropometric outcomes was a key finding of the present study. Correct early initiation of breastfeeding, colostrum feeding, and exclusive breastfeeding were associated with higher WAZ, while colostrum feeding and exclusive breastfeeding were associated with higher LAZ. These findings are consistent with international evidence that IYCF indicators are relevant to child growth, although the strength and direction of associations may vary across settings. Zongrone et al., using nationally representative data from Bangladesh, found that exclusive breastfeeding, dietary diversity, and minimum acceptable diet showed stronger associations with child growth outcomes than some other feeding indicators (21). Similarly, a multi-country synthesis by Jones et al. concluded that diet diversity and overall diet quality were positively associated with LAZ in several countries, while associations between breastfeeding indicators and anthropometry were more mixed (22).

The regression findings in the present study reinforce the importance of breastfeeding-related variables. In the WAZ model, early initiation of breastfeeding, exclusive breastfeeding, and total knowledge score were positively associated with WAZ, while number of children was negatively associated with WAZ. The negative association with number of children may reflect household resource dilution, reduced caregiver time, or greater competing demands for feeding and care. In the LAZ model, exclusive breastfeeding was positively associated with LAZ, while male sex showed a negative association. The positive association of exclusive breastfeeding with both WAZ and LAZ supports the importance of sustained breastfeeding promotion, although causality cannot be inferred from this cross-sectional design. Although several IYCF indicators were associated with WAZ and LAZ, these findings should not be interpreted as evidence of causality because exposure and outcome data were collected at the same time. Child growth is influenced by multiple factors, including birth weight, gestational age, morbidity, maternal nutritional status, household food security, and socioeconomic conditions. Therefore, residual confounding may partly explain the observed associations.

The knowledge–practice gap observed in this study is important for program planning. Although knowledge was generally high, several recommended behaviors were not practiced consistently. This finding suggests that educational interventions should move beyond simply communicating recommendations and should address practical barriers such as perceived insufficient milk, cesarean delivery, maternal fatigue, family influence, return to work, and misconceptions around infant satiety. The knowledge–practice gap observed in the present study is consistent with recent UAE-based maternal child-health evidence, where high overall vaccination uptake was reported, but delays, safety concerns, lack of awareness, and culturally influenced misconceptions remained important barriers (23). Saudi evidence has similarly highlighted that maternal knowledge, beliefs, and family influences can shape feeding practices among mothers of young children (24). Therefore, culturally sensitive counseling that includes mothers, fathers, and other family caregivers may be more effective than mother-focused education alone.

Delayed initiation of breastfeeding was most commonly attributed to perceived absence of breast milk secretion and cesarean delivery. This has direct implications for maternity care. Mothers who undergo cesarean delivery or report delayed milk secretion should receive early lactation support, reassurance, and practical assistance with positioning and attachment. Strengthening baby-friendly hospital practices, consistent postnatal counseling, and early follow-up may reduce unnecessary formula supplementation and improve continuation of exclusive breastfeeding (1, 6, 25).

From a public health perspective, the study findings indicate that improving IYCF practices in the Northern Emirates requires action at both facility and community levels. Antenatal counseling should prepare mothers for early initiation, exclusive breastfeeding, and common early breastfeeding challenges. Maternity and pediatric services should provide consistent messages on colostrum, avoidance of pre-lacteal feeding, and age-appropriate complementary feeding. For complementary feeding, messages should emphasize not only timing but also dietary diversity, meal frequency, iron-rich foods, fruits and vegetables, and avoidance of sweet beverages and unhealthy snacks. These recommendations are consistent with WHO guidance, which emphasizes adequate meal frequency and sufficient dietary variety for children aged 6–23 months (26).

### 4.1 Strengths and limitations

This study included both local and expatriate mothers from two Northern Emirates and assessed multiple dimensions of IYCF, including knowledge, practice, dietary indicators, and anthropometric outcomes. The use of an English and Arabic questionnaire increased accessibility for participants from different backgrounds. The inclusion of regression analysis also strengthened the epidemiological interpretation of feeding practices in relation to anthropometric outcomes.

However, several limitations should be acknowledged. First, the cross-sectional design limits causal interpretation. Second, feeding practices were self-reported and may be affected by recall bias or social desirability bias. Third, non-probability consecutive sampling limits generalizability to all mothers in the UAE. Because non-probability consecutive sampling was used and recruitment source was not recorded separately, selection bias cannot be excluded. Mothers recruited from pediatric outpatient settings may differ from mothers in the general community in healthcare-seeking behavior, child health status, and prior exposure to feeding counseling. In addition, the response rate could not be calculated because the number of eligible mothers approached and the number who declined participation were not systematically recorded. Anthropometric measurements in infants and toddlers are also technically challenging. Child movement, positioning during length measurement, clothing, and weighing conditions may have introduced minor measurement errors, particularly for length-for-age and weight-for-age z-scores. The internal consistency values for the knowledge and practice items were low to moderate. However, this was expected because the questionnaire included distinct IYCF indicators rather than items measuring a single latent construct. Although delivery term was recorded, it was based on maternal report and categorized only as full term or preterm. Exact gestational age, corrected age for preterm children, and birth weight were not collected; therefore, delivery term could not be used as a robust adjustment variable in the anthropometric regression models. Residual confounding related to birth characteristics cannot be excluded.

## 5 Conclusion

This study found generally favorable maternal knowledge of recommended IYCF practices among mothers of children under **2** years in Ras Al Khaimah and Fujairah. However, actual feeding practices were less optimal, particularly for exclusive breastfeeding, avoidance of pre-lacteal feeding, continued breastfeeding, and minimum acceptable diet. Several breastfeeding-related practices were associated with more favorable WAZ and LAZ, but these associations should be interpreted cautiously because of the cross-sectional design and possible residual confounding. Strengthening culturally appropriate facility-based and community-based counseling may help improve breastfeeding and complementary feeding practices in the Northern Emirates of the UAE.

## Data Availability

The original contributions presented in the study are included in the article/supplementary material, further inquiries can be directed to the corresponding author.
